# Changes in Activity of the Same Thalamic Neurons to Repeated Nociception in Behaving Mice

**DOI:** 10.1371/journal.pone.0129395

**Published:** 2015-06-12

**Authors:** Yeowool Huh, Jeiwon Cho

**Affiliations:** 1 Center for Neural Science, Korea Institute of Science and Technology, L7313 Hawolgok-dong Seongbuk-gu, Seoul, South Korea; 2 Neuroscience, University of Science and Technology, 217 Gajeong-ro, Yuseong-gu, Daejeon, South Korea; University of Texas at Dallas, UNITED STATES

## Abstract

The sensory thalamus has been reported to play a key role in central pain sensory modulation and processing, but its response to repeated nociception at thalamic level is not well known. Current study investigated thalamic response to repeated nociception by recording and comparing the activity of the same thalamic neuron during the 1^st^ and 2^nd^ formalin injection induced nociception, with a week interval between injections, in awake and behaving mice. Behaviorally, the 2^nd^ injection induced greater nociceptive responses than the 1^st^. Thalamic activity mirrored these behavioral changes with greater firing rate during the 2^nd^ injection. Analysis of tonic and burst firing, characteristic firing pattern of thalamic neurons, revealed that tonic firing activity was potentiated while burst firing activity was not significantly changed by the 2^nd^ injection relative to the 1^st^. Likewise, burst firing property changes, which has been consistently associated with different phases of nociception, were not induced by the 2^nd^ injection. Overall, data suggest that repeated nociception potentiated responsiveness of thalamic neurons and confirmed that tonic firing transmits nociceptive signals.

## Introduction

A body could be involuntarily exposed to repeated injuries, but how the body responds to repeated injury is poorly understood. Studies on repeated tissue injuries suggest that it may lead to either adaptive or pathological changes. Protective adaptation was reported to occur in some cases with decreased inflammatory response and increased tissue regeneration [1–4], while another study reported reduced tissue regeneration and increased responsiveness of primary afferent neurons, suggested to be a trigger for developing chronic pain [5]. Despite many studies that tie abnormalities in thalamic structure and activity with various chronic pain states ranging from arthritis to neuropathic pain [6–8], understanding of neuronal response to repeated pain at thalamic level is yet limited.

The thalamus is a structure which relays sensory information to the cortex including pain signals [9]. The sensory thalamus, which includes the ventral posterolateral (VPL) and the ventral posteromedial (VPM) nuclei, has nociception specific neurons and is hypothesized to play a key role in sensory signal discrimination and modulation [10–15]. Studies showing alteration in thalamic structures or changes in thalamic activities associated with many different types of pain disorders emphasize its importance in pain signal processing [7,8,16,17]. Sensory modulation is proposed to occur via the characteristic ability of a single thalamic neuron to switch between tonic and burst firing modes [15,18,19], respectively referring to firing in single spikes or a burst of high frequency spikes. Since the switch between the two firing modes are dependent on arousal state with burst firing becoming more prevalent during sleep or anesthesia [20–22], some studies suggested that increased thalamic burst firing activity observed during the awake state of patients may cause neuropathic pain [8,17,23]. Whether increased burst firing during the awake state is actually the cause of neuropathic pain is controversial [16,24], but alterations in burst firing properties have beenreported in neuropathic pain models [25,26].

Although many studies investigated the role of the thalamus in acute and chronic pain conditions, how thalamic neurons respond to repeated nociceptive stimuli is not well studied. Considering the importance of the thalamus in pain processing, response of thalamic neurons to repeated nociceptive stimuli has been investigated in this study. Activities of identical neurons in the sensory thalamus, that directly receives nociceptive inputs via the spinothalamic tract [9], were recorded before and after two nociceptive stimulations induced by formalin in awake and behaving mice. Formalin induced nociception was used because it is a well characterized pain model suggested to be a good model of clinical pain [27].

## Materials and Methods

### Ethics statement

All experiments were approved and conducted in accordance to the guideline of Animal Care and Use Committee (IACUC) of Korean Institute of Science and Technology (protocol number: AP-201001034). Surgical procedures were conducted under general anesthesia (Zoletil, 30 mg/kg body weight) and monitored daily after surgery. Mice were handled gently before and during experiments to minimize their suffering.

### Subjects

First generations of C57BL/6J × 129/SvJae hybrid male mice were used in experiments. Mice were maintained at constant temperature (22±1°C) with free access to food (constant nutrition formula, PicoLab) and water under a 12:12 hour light and dark cycle (light cycle beginning at 8:00 AM). Behavioral and neuronal recordings were done in different sets of mice due to technical constrains, since recording cable attached to microdrives for neuronal recordings interfered with the expression of nociceptive behavior such as licking. However, general movements were unhindered and mice were freely moving with the recording cable attached. Mice for behavior assessment were group caged: 2–3 mice per cage. For neuronal recordings, mice were initially group caged, but were individual caged after microdrive implantation surgery to protect implantation.

### Behavioral assessment of nociceptive responses

Total of 9 mice (10–14 weeks, 24–33g body weight) were used for behavioral assessment of repeated nociception. Five mice (11–12 weeks, 24–25g body weight) were used for control. Prior to all tests, mice were handled and habituated to the recording environment for at least 20 min per day for a week. Recording chamber were made of opaque plastic cylinder (20 cm diameter, 25 cm height) placed on top of a clear plastic cube with a beveled mirror placed at a 45° angle for behavioral monitoring. To induce nociception, 10 μl of formalin (5%, 1:20 dilution of 37% formalin solution in deionized water) was injected into the left hind plantar paw and behavior was videotaped for an hour. Second formalin injection was given after a week. For control, saline (10 μl) was injected in place of formalin using the same protocol. After completion of behavioral experiments, videos were analyzed by at least two investigators ‘blinded’ to test groups. Degree of nociception was measured by summing the duration of licking and shaking behavior of the formalin injected paw in 5 min segments. Then phases-wise analysis was done based on behavioral nociception changes for better comparison between behavior and neuronal activities.

### Microdrive implantation surgery

Craniotomy was performed to record neural signals. Mice were anesthetized with Zoletil (30 mg/kg body weight, i.p.) and supplementary dose, one third of the initial dose, was given to maintain full anesthesia throughout the surgery. Anesthetized mice were hooked up onto a stereotaxic instrument (Kopf, USA) for craniotomy. A hole was drilled above the target after exposing the skull and a microdrive with tetrodes (four 12.5μm nichrome polyamide-insulated microwires were intertwined into one tetrode, Kanthal precision technology; recording tip of each tetrode channel was gold plated to 400–500 kΩ) was placed into the right sensory thalamus (VPM/VPL; AP: -1.57 mm, ML: -1.8 mm, DV: -3.25 mm). After implantation, the microdrive was secured onto the skull with stainless steel screws and dental cement. Mice were allowed to recover for a week and the condition of mice was monitored every day.

### Extracellular single unit recording

Recordings were done under dim lighting with a white noise generator set at a maximum of 85 dB. Each mouse was allowed to habituate to the experimental setting, identical to the one used for behavioral assessment, for at least 20 min. Neuronal signals were obtained with the Cheetah Acquisition System (Neuralynx, USA) and signals were filtered, amplified, and sampled at 30,303 Hz. For both formalin injections and saline control, spontaneous firing rate before injection was recorded for 10 min as baseline and induced firing rate after injection was recorded for an hour. A week interval was given between formalin injections. Injections (10 μl of saline or 5% formalin, 1:20 dilution of 37% formalin solution in double de-ionized water) were given to the left plantar paw, contra-lateral to the neuronal signal recording side. Mice were unrestrained and allowed to freely move during all recording sessions.

### Data analysis

Neuronal spike data were collected with the Cheetah Acquisition System (Neuralynx, USA) and sorted into single units using the SpikeSort3D program (Neuralynx). Isolated signals were confirmed to originate from a single unit with inter-spike-interval histograms and cross-correlation analysis. Only the same pairs of well-isolated signals recorded during both the 1^st^ and 2^nd^ formalin injections, confirmed to be in the sensory thalamus post mortem, were used for data analysis. Thalamic neuronal signals were obtained from total of 9 mice, but only 5 mice (10-14weeks, body weight 24–32 g) had signals from identical neurons in both formalin injections. For saline control, all recorded neurons (not identical neurons recorded during both saline injections) obtained from 4 mice (12–13 weeks, body weight 24-26g), were analyzed. Overall, tonic and burst firing rates (spikes/s) were analyzed from spike train of individual neurons in 5 min segments or in phases (phase I, interphase, phase II, and phase III) for better comparison between neuronal signals and behavior. Since a single thalamic neurons is able to switch between tonic and burst firing, tonic and burst spikes were separated from overall spikes by distinguishing burst spikes and considering all non-burst spikes to be tonic spikes. A criterion for distinguishing low threshold spike (LTS) bursts [28]—spikes consisting of at least 2 spikes occurring within ≤4 ms with ≥100 ms proceeding silence—was used to determine burst spikes. Bursting properties—the number of burst spikes composing a burst, interval-between-bursts, and interval-between-burst-spikes—were also analyzed over time in 5 min segments. To compare changesin firing rate induced by formalin or saline injections, relative to the baseline firing rate, data were normalized by the following method: (firing rate after injection–baseline firing rate) / (firing rate after injection + baseline firing rate). This normalization method gives an accurate representation of the neural changes induced by formalin relative to the baseline of each individual cell, but the magnitude of relative neuronal activity changes is not reflected. In this method, value of -1 is the minimum, +1 is the maximum, and 0 indicates no change. Two tailed t-test was used to test the difference in firing rate and burst firing properties between the 1^st^ and 2^nd^ formalin injections and to compare changes induced by the 2^nd^ formalin or 2^nd^ saline injection with the baseline. A p-value of 0.05 was used to determine significance.

### Histology

Locations of neuronal signal recording were verified with histology. Small electrolytic lesion was made at the tip of the recording site by passing anodic current (5–20 μA, 10 s) through tetrode channels. After completion of the study, mice were overdosed with 2% avertin and transcardially perfused with saline (0.9%) followed by formalin (10% formalin diluted in saline). Brains were removed and stored in formalin (10% formalin diluted with deionized water) for a day, and transferred to a 30% sucrose solution for another day for further fixation. Fixed brain tissues were cut frozen in coronal sections (50 μm) through the entire thalamus with a microtome (Microm). Brain slices were dried for a day and stained with cresyl violet. Stained slices were examined under a light microscope to determine locations of electrolytic lesion.

## Results

### Behavioral response to repeated nociception

**Fig 1:**
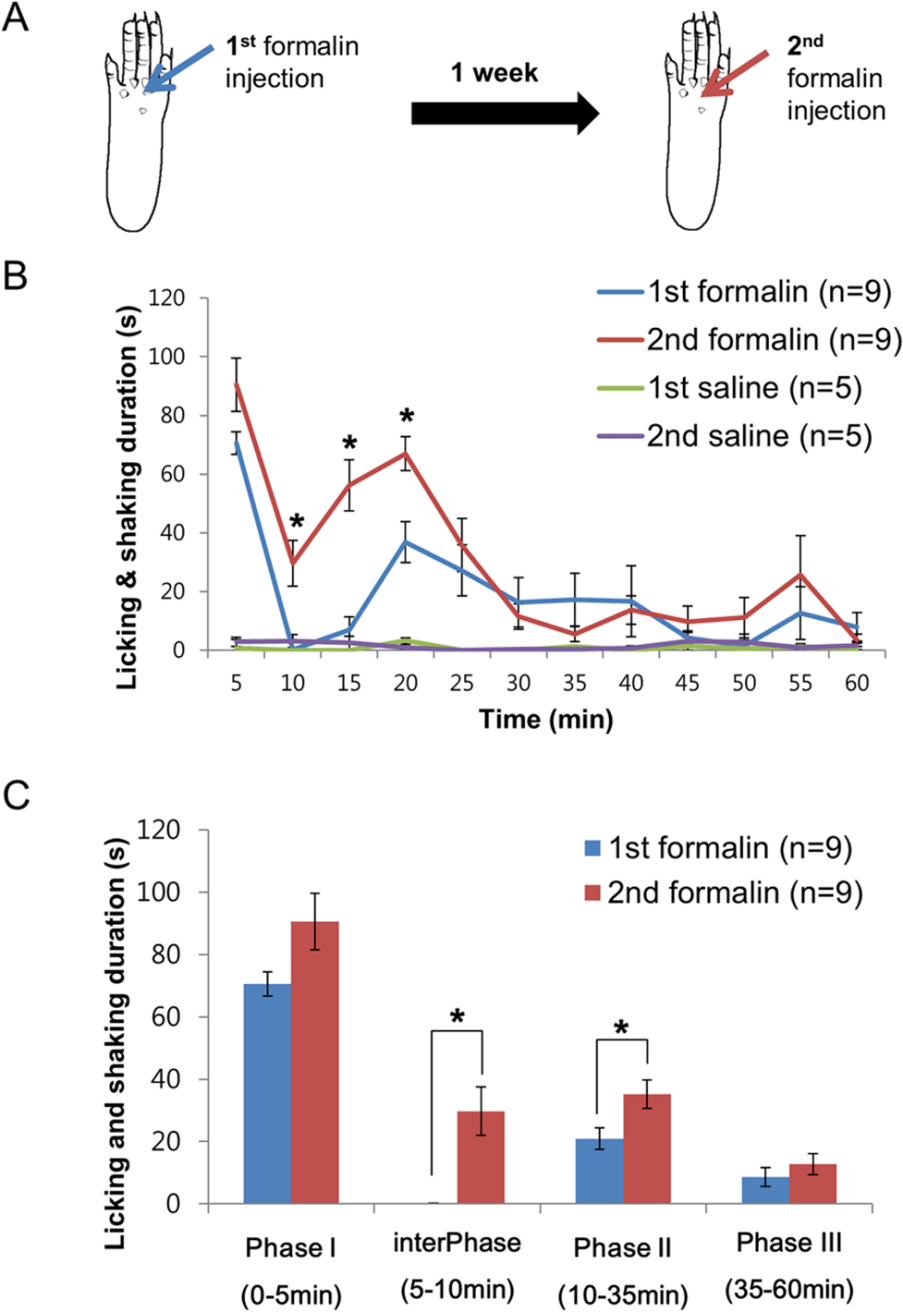
Behavioral response to repeated nociception. **(A)** Schematic drawing of the experiment procedure. Formalin or saline was injected into the left paw pad twice with one week term. **(B)** Behavioral nociceptive response of 1^st^ and 2^nd^ formalin or saline injections. **(C)** Phasic analysis of behavioral nociceptive responses of 1^st^ and 2^nd^ injections, based on changes in behavior. All data points are mean±SEM. Two tailed t-test was used to compare the degree of nociception between 1^st^ and 2^nd^ formalin injections for each time segments and phases or between 1^st^ and 2^nd^ saline injections at each time segments, *P<0.05.

First, behavioral response to repeated nociception was investigated by injecting formalin (5%, 10 μl each injection) or saline (10 μl) twice to the plantar hind paw of mice with a week term (Fig 1A). Both the 1^st^ and 2^nd^ formalin injections induced characteristic response of a formalin test with phasic nociceptive responses separated by the interphase (5–10 min) with identical peak nociceptive response timing at 0–5 min and 15–20 min (Fig 1B). Saline injections, however, did not trigger any nociceptive responses and there were no response difference between the 1^st^ and 2^nd^ saline injections (Fig 1B). Since the formalin pain model triggers complex changes in behavior, nociceptive responses were also analyzed in phases, based on the rise and declination in nociceptive behavior, for direct and simplified comparisons between behavior and neuronal activities: phase I (0–5 min), interphase (5–10 min), phase II (10–35 min), and phase III (35–60 min; Fig 1C). The 2^nd^ formalin injection induced hyperalgesic response relative to the 1^st^, with significantly higher degree of nociception during the interphase and initial part of the phase II nociception (5–20 min; Fig 1B). PhaseI of the 2^nd^ formalin injection had a tendency to be slightly higher than that of the 1^st^ formalin injection, but was statistically insignificant (*P* = 0.06, Fig 1B). These differences in the degree of nociception between the 1^st^ and 2^nd^ injections are well represented in the phasic analysis of behavior, showing significant difference in the interphase and phase II (Fig 1C).

### Relationship between thalamic activity and repeated nociception

**Fig 2:**
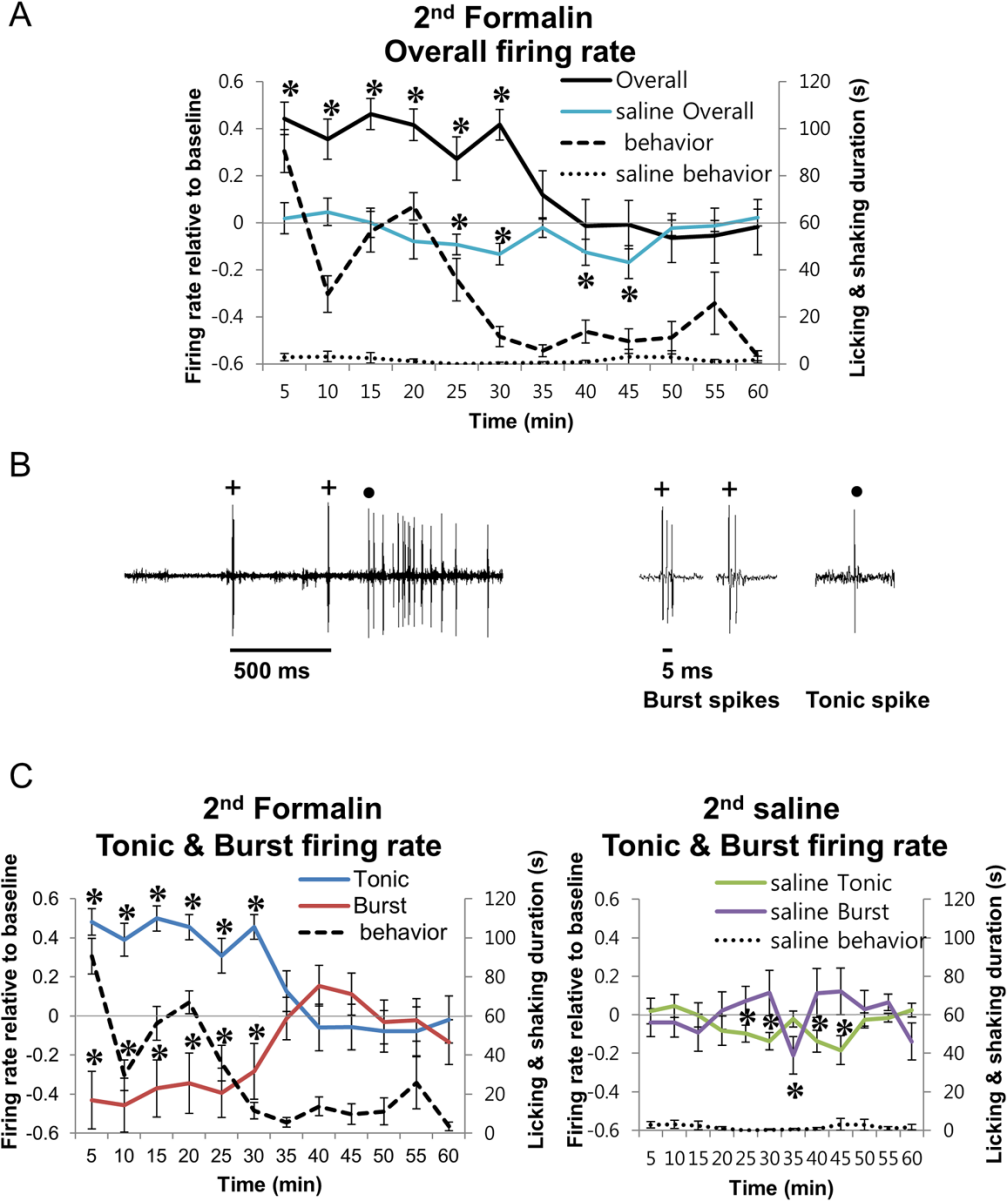
Thalamic neuronal activity changes induced by 2^nd^ formalin or 2^nd^ saline injection. **(A)** Overall thalamic neuronal firing rate changes and behavior nociceptive responses induced by 2^nd^ formalin or saline injection. **(B)** Sample of a thalamic neuron firing in burst (+) or tonic (•) spikes. **(C)** Tonic and burst firing of thalamic neurons after 2^nd^ formalin or saline injection. Blue line indicates tonic firing, red line indicates burst firing, and dotted line is the behavioral responses. **(A and C)** All data points are mean±SEM. Formalin *N* = 19 neurons from 2^nd^ injection recorded in pairs, 5 mice. Saline *N* = 13 neurons, 4 mice. Two tailed t-test was used to compare each data points with respective baselines. * indicates significant difference at *P*<0.05.

Next, relationship between thalamic activity and behavior induced by the 2^nd^ formalin injection was investigated to show whether changes in thalamic activity reflect behavioral changes. In total, activities of 48 and 34 single neurons were recorded during the 1^st^ and 2^nd^ formalin injections, respectively, but only 19 neurons recorded during both injections were used in the current analysis. All 13 neurons recorded during the 2^nd^ saline injection were analyzed because there were no significant behavioral differences between the 1^st^ and 2^nd^ saline injections. Activities of primary sensory thalamic neurons recorded in the VPM and VPL region were normalized relative to the baseline to reveal changes induced by injections. Changes in overall firing rate of thalamic neurons by formalin injection mirrored the changes of the behavioral nociceptive responses. It significantly increased relative to the baseline during phase I, interphase, and most of phase II when behavioral nociceptive responses remained high (0–30 min) and then returned to the baseline to remain at baseline level during phase III (35–60 min; Fig 2A). Overall firing rate of the 2^nd^ saline injection, on the other hand, never increased above the baseline level (Fig 2A saline Overall). Since a single thalamic neuron is able to switch between burst and tonic firing modes, which are suggested to have differential roles, burst and tonic firing were separated from overall firing as described in the Methods section and analyzed. Samples of a single neuron’s firing in burst and tonic mode are shown in Fig 2B. Tonic and burst firing activity also changed interactively according to the changes in behavioral nociceptive responses. Tonic firing increased significantly relative to the baseline after the 2^nd^ formalin injection, like that of the overall firing rate, while burst firing was significantly decreased during the same period (Fig 2C). Both tonic and burst firing activity returned to the baseline level by phase III, when behavioral nociceptive responses were low. In contrast, no changes in tonic and burst firing rate changes were induced by the 2^nd^ saline injection at most time segments.

**Fig 3:**
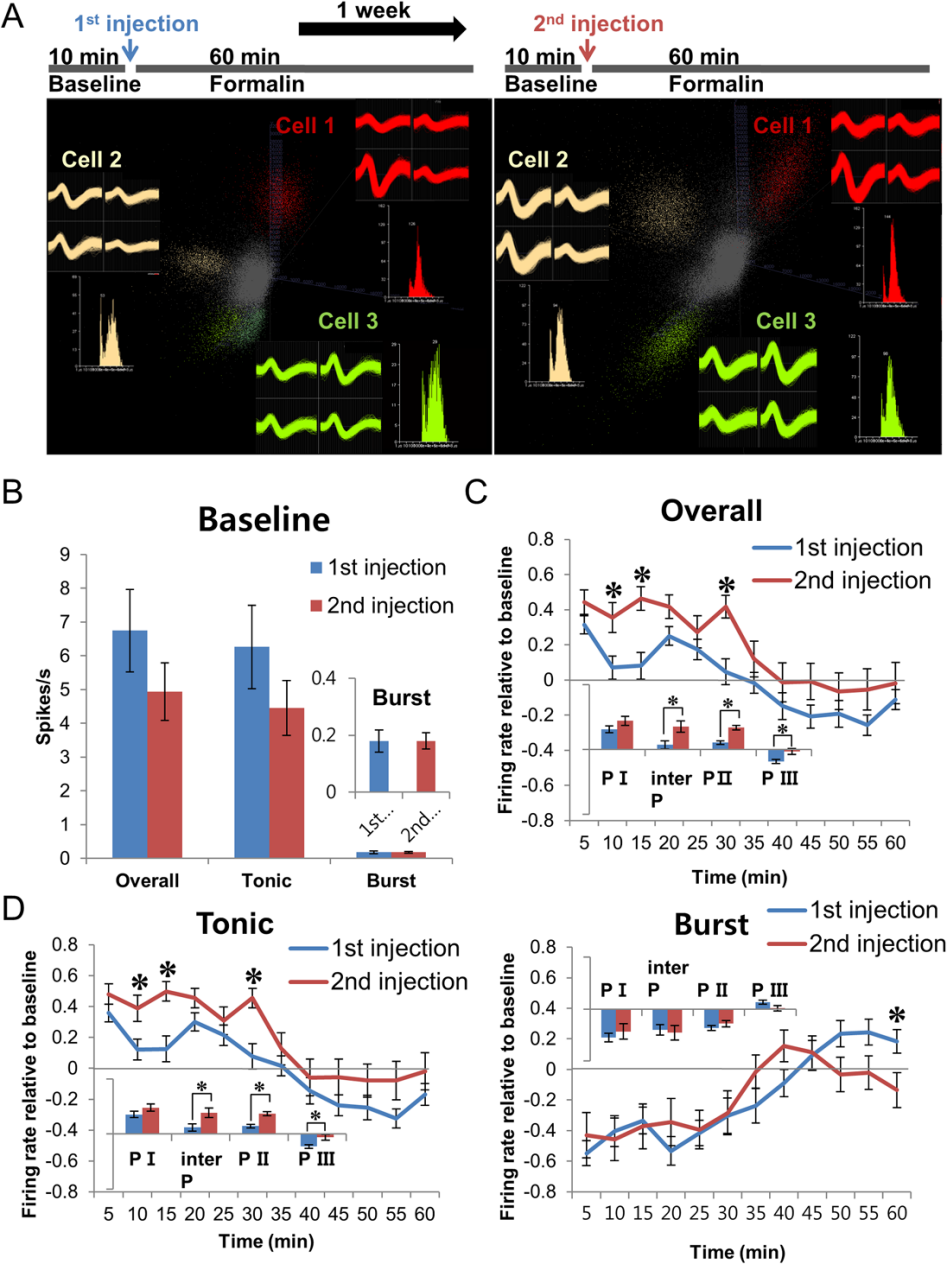
Paired comparison of thalamic neuronal activity changes during the 1^st^ and 2^nd^ formalin injection. **(A)** Outline of experimental procedures and cluster cut sample of the same cells recorded during the 1^st^ and 2^nd^ formalin injection. **(B)** Comparison between baseline firing rates before 1^st^ and 2^nd^ formalin injection. **(C)** Overall thalamic neuronal firing rate changes induced by the 1^st^ and 2^nd^ formalin injection. **(D)** Tonic and burst firing rate changes of thalamic neurons induced by 1^st^ and 2^nd^ formalin injection. **(B-D)** All data points are mean±SEM of normalized value relative to respective baselines (see Methods).*N* = 19 pairs of the same neurons during 1^st^ and 2^nd^ injection, 5 mice. PI, interP, PII and PIII of bar graphs respectively indicate phase I (0–5 min), interphase (5–10 min), phase II (10–35 min), and phase III (35–60 min), based on changes in behavioral nociception. Two tailed t-test was used to compare each data points between 1^st^ and 2^nd^ injection. * indicates significant difference at *P*<0.05.

To track changes in thalamic response to repeated formalin injections, the same neurons recorded during both injections were analyzed in pairs (19 pairs). Fig 3A shows spike sorting sample of the three paired neurons recorded during both injections. The same neuron was identified by similar topology of waveform shapes and sizes captured from individual wires of a tetrode and the shape of an inter-spike-interval histogram. As shown in the spike sorting samples, some neurons that were detected during the 1^st^injection disappeared and new neurons appeared during the 2^nd^injection, but these neurons were excluded from the analysis. Baseline neuronal firing frequencies before formalin injections were the same for overall, tonic, and burst firing between injections (Fig 3B). However, the 2^nd^ formalin injection induced significant potentiation of the overall thalamic firing rate, compared to that of the 1^st^, during all phases except phase I (Fig 3C, bar graph), indicating that the 2^nd^ injection induced potentiation of thalamic firing relative to the baseline than the 1^st^. Potentiated tendency of nociceptive behavior during phaseI (*P* = 0.17) was also reflected in the overall thalamic activities. In sum, potentiation of behavioral nociception was reflected by potentiation of thalamic activity. Additional analysis of tonic and burst firing,showed that changes in tonic firing rate were the same as thechanges in the overall firing rate with significant potentiation induced by the 2^nd^ injection in all phases except for phase I (Fig 3D, Tonic). Burst firing rate changes, however, were similarbetween both injections in all phases without any significant differences (Fig 3D, Burst bar graph). Changes in firing rate and relative ratio of tonic and burst spike firing during different phases of nociception are summarized in Table 1. The table confirms the response pattern and shows that majority of thalamic activities recorded during the awake state are tonic spikes.

**Table 1:** Comparison of Thalamic Discharges to the 1^st^ and 2^nd^ Formalin Induced Nociception.

		1st injection		2nd injection	
		Tonic	Burst Spike	Tonic	Burst Spike
**Baseline**	**FR(spikes/s)**	6.26±1.24	0.48±0.11	4.46±0.81	0.48±0.08
**(-10~0min)**					
	**ratio (%)**	92.5	7.5	89.6	10.4
**Phase I**	**FR(spikes/s)**	11.10±1.28	0.09±0.02	12.37±1.16	0.08±0.02
**(0-5min)**					
	**ratio (%)**	99.2	0.8	99.3	0.7
**interPhase**	**FR(spikes/s)**	7.96±1.53	0.14±0.04	10.29±1.36	0.12±0.04
**(5-10min)**					
	**ratio (%)**	98.2	1.8	98.8	1.2
	**FR(spikes/s)**	8.53±0.62	0.19±0.03	10.93±0.65	0.18±0.02
**Phase II**					
**(10-35min)**	**ratio (%)**	97.6	2.4	98.1	1.9
	**FR(spikes/s)**	4.97±0.57	0.72±0.09	5.04±0.48	0.51±0.04
**Phase III**					
**(35-60min)**	**ratio (%)**	87.1	12.9	90.5	9.5

Baseline is the spontaneous neuronal activity before formalin injection. Neuronal response after formalin injection is divided into phases based on changes in the behavioral pain responses. *N* = 19pairs of neurons from5 mice. All values are mean±SEM. FR: firing rate, ratio: % of respective firing modes from the total number of spikes.

### Individual neuronal response types to repeated nociception

**Fig 4:**
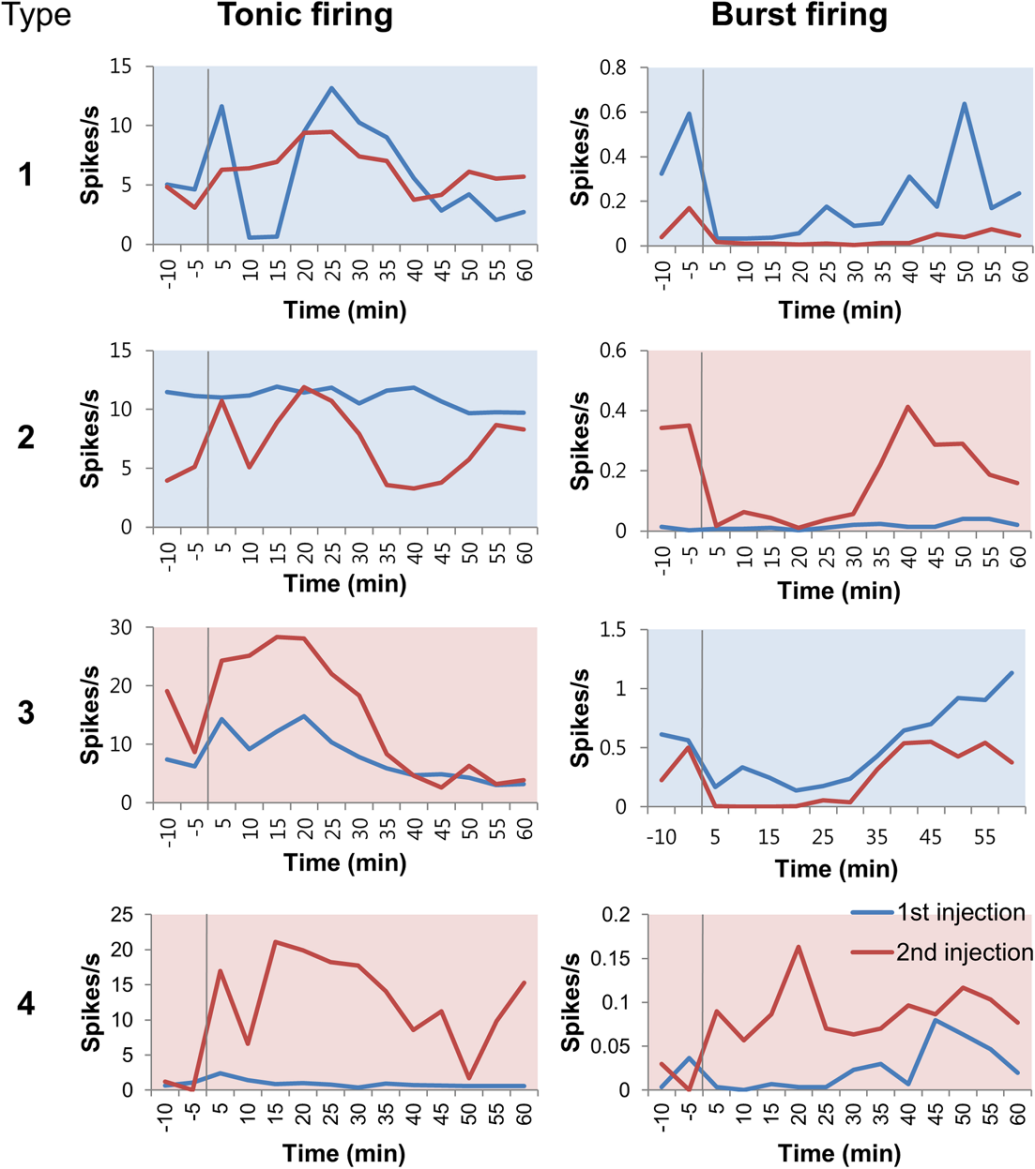
Types of thalamic neuronal activity changes induced by 1^st^ and 2^nd^ formalin injection. Example of four different types of neuronal activity changes of the same neuron for 1^st^ and 2^nd^ formalin injection is shown. Tonic firing activities are on the left and burst firing activities are on the right. All blue lines are data during the 1^st^ formalin injection, while all red lines are data during 2^nd^ formalin injection. Vertical grey lines indicate the point of formalin injection. Plots with blue backgrounds indicate cases where firing rate of 1^st^formalin injection was higher than that of the 2^nd^. Plots with pink backgrounds indicate the reverse. Sample shown for type 4 is also a neuron that had prominent theta oscillation after 2^nd^ injection.

Analysis by averaging may mask individual thalamic response patterns, thus, individual neuronal responses before and after the two formalin injections were examined. Four types of representative neuronal responses are delineated in Fig 4. Type 1 includesneurons that had lower tonic and burst firing rate in the 2^nd^ injection than the 1^st^ (3 out of 19). Type 2 includes neurons that had lower tonic firing, but higher burst firing in the 2^nd^ than the 1^st^ (3 out of 19). Type 3 had higher tonic firing, but lower burst firing in the 2^nd^ injection than the 1^st^ (6 out of 19). Type 4 neurons had higher tonic and burst firing in the 2^nd^ injection than the 1^st^ (7 out of 19). Most cells belonged to type 3 or 4, signifying that most thalamic neurons increased tonic firing, but had mixed responses in burst firing rate changes duringre-exposure to nociception.

### Burst firing properties and repeated nociception

**Fig 5:**
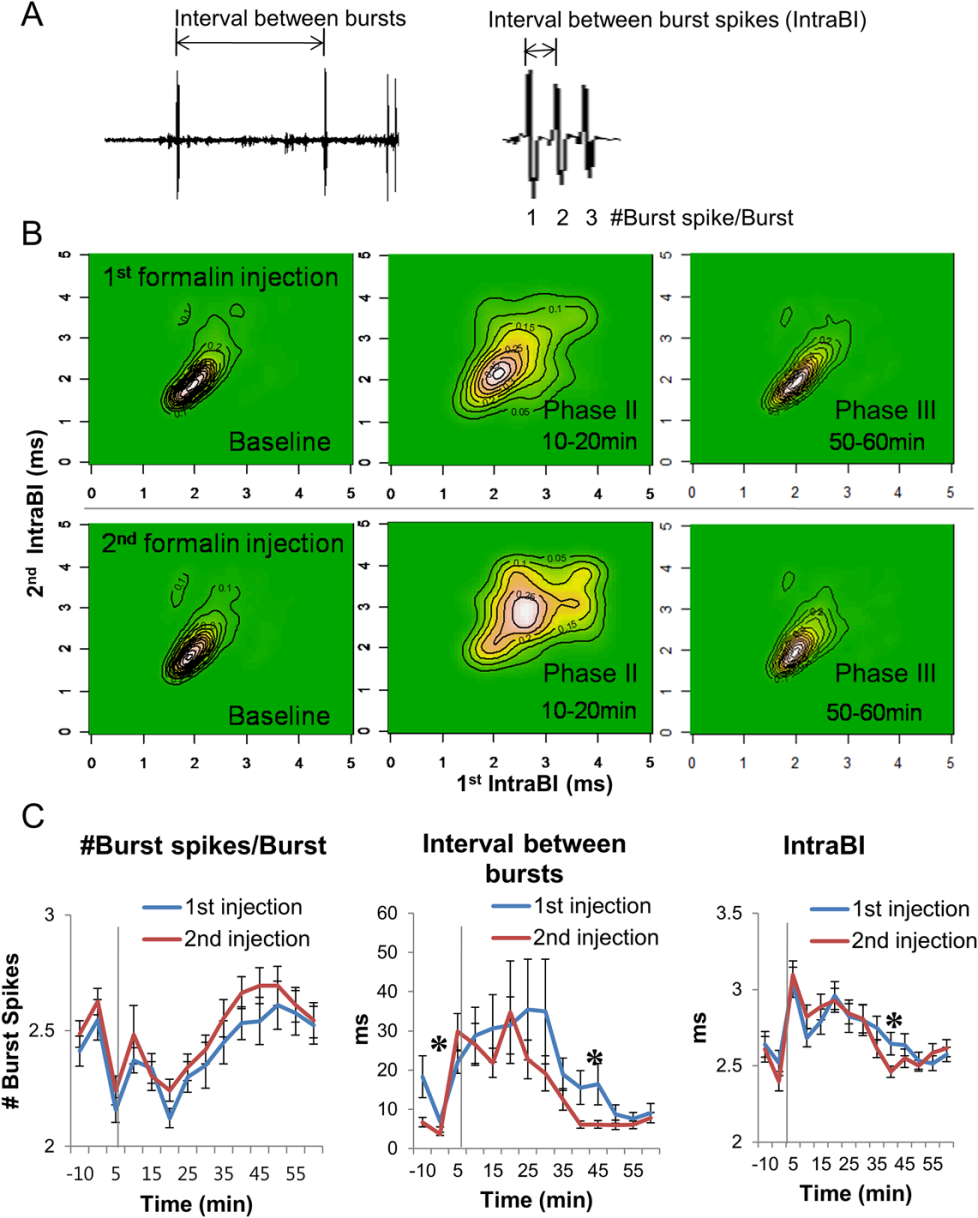
Thalamic burst firing property changes induced by the 2^nd^ formalin injection. **(A)** Parameters used in the burst firing property analysis. **(B)** Joint probability density analysis between 1^st^ and 2^nd^IntraBI of bursts at different time segments. **(C)** Burst firing property changes induced by 1^st^ and 2^nd^ formalin injection. Vertical grey lines indicate the point of formalin injection. All data points are mean±SEM. *N* = 19 pairs of same neurons during 1^st^ and 2^nd^ injection, 5 mice. Two tailed t-test was used to compare each data points between 1^st^ and 2^nd^ injection. * indicates significant difference at *P*<0.05.

Thalamic burst properties were shown to change accordingly to different phases of formalin induced nociception [15] and altered under neuropathic pain [25]. Thus, differences in burst firing properties between two formalin injections have been investigated as delineated in Fig 5A. Joint probability density (JPD) has been computed between the first and second intervals of burst spikes within a burst, namely Intra-Burst-Interval (IntraBI), IntraBI1 and IntraBI2 (Fig 5B). JPD is the probability distribution between the length of IntraBI1 and IntraBI2, showing integrity of burst spikes within a burst. Before formalin injection and during phase III, whenbehavioral nociceptive response is low, JPDs of both the 1^st^ and 2^nd^ formalin injections were similar. However during phase II, when nociceptive responses are high, burst integrity during the 2^nd^ injection was lower than the 1^st^ and widely dispersed. This signifies that probabilistically corresponding lengths of the IntraBI1 and IntraBI2 became more variable after the 2^nd^ injection compared to the 1^st^. However, there were no differences between the two injections in averages of burst parameters (Fig 5C). The mean number of burst spikes composing a burst was the same. The averages of interval-between-bursts and IntraBI between injections were also similar except at one segment (35–40 min), where the burst firing activity after the 2^nd^ had a tendency to be slightly greater than the 1^st^. Most burst properties did not differ between the two injections, implicating that temporal integrity of consecutive IntraBIs may be more critical in gating pain signals.

### Oscillation and repeated nociception

**Fig 6:**
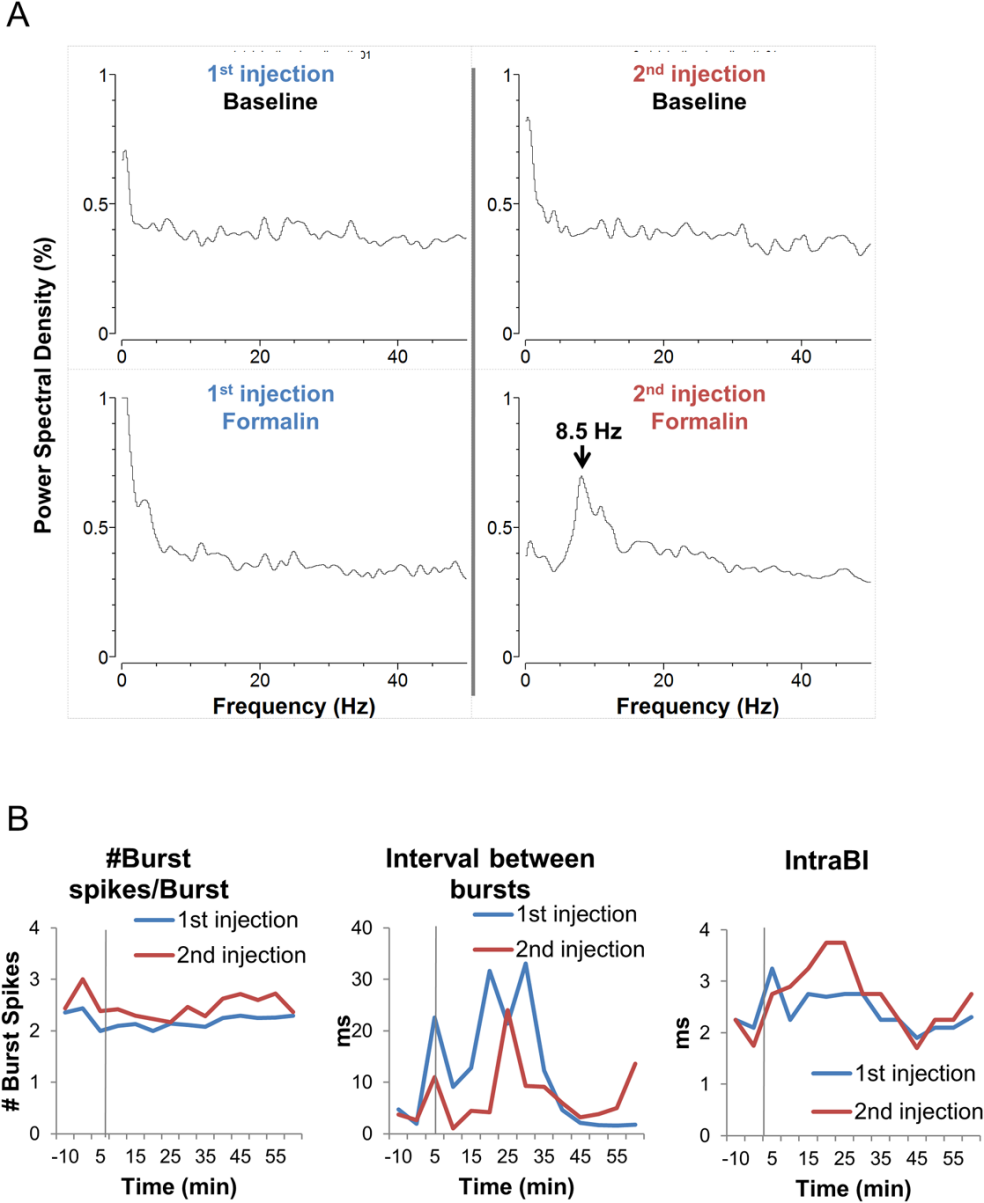
Analysis of a neuron that exhibit theta frequency oscillation after the 2^nd^ formalin injections. **(A)** Left top and bottom is a power spectral density before and after the 1^st^ formalin injection, respectively. Right top and bottom is a power spectral density before and after the 2^nd^ formalin injection, respectively. **(B)** Burst firing property analysis of the same neuron for 1^st^ and 2^nd^ formalin injections. Vertical grey lines indicate the point of formalin injection.

Multiple studies showed that chronic pain patients had increased power in the theta frequency band compared to controls. Whether the 2^nd^ formalin injection induced any changes in cellular oscillation frequency had been analyzed with power spectrum analysis in all recorded cells. All neuronal recordings were done in the awake and behaving state, in which oscillations are unlikely in non-pathological conditions. Unlike the 1^st^ formalin injection where no oscillation was observed in any cells (0 out of 48 cells), 2 cells out of 34 (6%) cell fired in rhythmic oscillation even in the awake stateafter the 2^nd^ formalin injection. Both cells had maximum power at 8.5 Hz, which is within the theta frequency range (4–9 Hz), and had peak power spectral density (PSD) value of 0.50% and 0.72% (Fig 6A). Of the two cells showing oscillation, one was recorded during both injections (1 out of 19 pairs of cells, 5%). This cell had a power spectrum mostly in the very low frequency (<1 Hz) during the baselines before injections, and after the 1^st^ formalin injection. However, the 2^nd^ formalin injection induced prominent oscillation in this cell with maximum power at 8.5 Hz (PSD 0.72%). This cell had type 4 neuronal activity changes, which is the cell shown in Fig 4, with increased tonic and burst firing activities due to the 2^nd^ formalin injection compared to the 1^st^. Since this cell may have different burst firing properties from those of the other cells, burst firing properties of this cell was analyzed using the same parameters described in Fig 5A. Indeed this cell had different burst firing properties between the two injections (Fig 6B). The average number of burst spikes composing a burst was consistently greater in the 2^nd^ injection. The interval-between-bursts was shorter in the 2^nd^ injection at most time segments after formalin, indicating increased burst firing frequency by the 2^nd^ injection. The IntraBI, however, was longer during the interphase and early phase II (5–25 min) after the 2^nd^ injection, which is when behavioral nociception of the 2^nd^ injection was greater than the 1^st^.

## Discussion

Results show that thalamic neurons increased firing to repeated nociceptive stimulation in accordance with potentiated behavioral nociceptive responses. In terms of tonic and burst firing, tonic firing was potentiated while there were no changes in burst firing activities by the 2^nd^ injection, confirming the interactive role of tonic and burst firing in processing nociceptive information during the awake state [15].

Behaviorally, nociceptive response of the interphase and phase II was significantly elevated by the 2^nd^ injection. This indicates that phase II of the formalin test, thought to occur by the development of inflammation, was affected more than phase I, suggested to occur mainly by direct activation of nociceptors [29]. Behavioral hypersensitivity to the 2^nd^ injection is probably due to lasting hyperalgesia following the 1^st^ injection because mechanical and thermal hyperalgesia is reported to last for more than 3 weeks [30]. Lasting hyperalgesia may have also reduced the efficiency of peripheral inhibition, since the interphase is suggested to occur by active peripheral inhibition [31]. Despite the significant potentiation induced by the 2^nd^ injection, nociceptive responses rapidly reduces to the level of the 1^st^ injection after the peak of phase II. Since greater degree of nociception reduction must occur in the 2^nd^ injection to reach the nociception level of the 1^st^, adaptive changes may have occurred after the 1^st^ injection to effectively reduce the potentiated nociceptive responses of the 2^nd^.

Although rapid reduction of potentiated behavioral responses after phase II insinuates the possible presence of adaption to repeated nociception, no adaptive changes were evident in the sensory thalamic responses. Rather, overall thalamic activity was potentiated by the 2^nd^ injection. Thalamic sensitization was shown to occur by inflammatory mediators inducing allodynia [32] and inflammation-produced hyperalgesia was shown to be mediated by thalamic NMDA receptor activation [33]. Given the nature of formalin induced nociception, which is due to the development of inflammation during phase II, thalamic sensitization mediated by NMDA receptors may have occurred by the repeated injections. However, sensitizations of the periphery and the spinal cord might also be reflected in the potentiated thalamic response.

Differential analysis of tonic and burst firing showed that tonic firing also was significantly potentiated in accordance to the potentiated behavioral nociceptive responses. Tonic and overall firing patterns were similar because tonic firing was the predominant firing mode during the awake state, composing at least 87% of all spikes in the present study. Positive relationship between tonic firing and behavioral nociception in both injections demonstrate an agreement with the hypothesis that tonic firing serves as a faithful representation of the external world [18]. Since the sensory thalamus also receives tactile inputs [9], tactile information induced by licking of the inflicted paw may also be reflected in tonic firing changes. In contrast to tonic firing activity changes, no changes were observed in burst firing activities between injections. This reduction may be due to dichotomy of neuronal populations that either increased (10 out of 19 cells) or decreased burst firing (9 out of 19 cells) almost in equal sample size by the 2^nd^ injection compared to the 1^st^, driving the average value towards no change. Unlike differential thalamic responses induced by repeated nociception, baseline tonic and burst firing activities before formalin injections did not differ, indicating that there were no lasting changes in spontaneous firing activity induced by the 1^st^ injection.

Focusing on the changes in burst firing properties associated with neuropathic pain [25,26], possible alteration that may have occurred by repeated nociception was investigated and found no significant differences between injections. Decrease in the average interval-between-bursts and IntraBI after the 2^nd^ injection at one time segment (35–40 min) may be caused by slight higher, despite the statistically insignificant, relative changes in burst firing rate of the 2^nd^ injection compared to that of the 1^st^ during that period. Increased burst firing frequency will shorten the interval-between-bursts and decrease the IntraBI. Normal level of burst activity can modulate potentiated nociception and another implication is that no neuropathic condition has yet developed by the 2^nd^ formalin injection. However, the dispersed shape of the JPD analysis between IntraBI1 and IntraBI2 induced by the 2^nd^ injection in phase II implies that integrity of consecutive burst spikes composing a burst may have been compromised by the 2^nd^ injection in a way that is less effective in reducing nociception. This may have led to greater behavioral nociception during the phase II, since previous studies demonstrated that the length of IntraBI is significant in producing an anti-nociceptive effect [15,34].

Oscillation study showed that most cells did not have any oscillation during both injections. However, two thalamic neurons developed prominent oscillation in the theta frequency after the 2^nd^ injection, even though the recording was done in the awake state. There were no single neuron that exhibited any oscillations to the 1^st^ formalin injection (48 neurons, unpublished data), strongly suggesting that the 2^nd^ injection induced oscillation in these neurons. Increase in theta frequency oscillation in the thalamus has been observed in many forms of pathological conditions including neuropathic pain [10,35–38]. This power increase in theta frequency has been proposed to be due to increase in burst firing activity [37]. Consistently, the one neuron that was recorded in pairs and developed oscillation in our data had increased burst firing by the 2^nd^ injection compared to the 1^st^, although not all neurons with increased burst firing activity showed oscillation. In addition, burst firing properties were found to be altered. The number of burst spikes composing a burst and the burst firing frequency increased, but integrity of burst spikes, shown by the IntraBI, decreased by the 2^nd^ injection, during the interphase and phase II, which corresponds to the phase of increased behavioral nociception. As already mentioned, changes in burst firing properties have been associated with neuropathic pain [25] and loosened integrity of burst spikes composing a burst directly influenced the ability of burst firing to have an anti-nociceptive effect [34]. Increase in the number of cells responding in this pattern, even in the awake state, may eventually lead to the development of chronic pain. If so, repeated formalin injection may be used as a transitional model that links acute and chronic pain. However, case size was too small to definitely determine this in the current study and more injections may be necessary to develop into chronic pain.

Overall, our data show that sensory thalamic neurons increased responsiveness to repeated nociception and confirmed the interactive roles of tonic and burst firing in processing nociceptive information.
